# Pretreatment platelet count predicts survival outcome of patients with *de novo* non-M3 acute myeloid leukemia

**DOI:** 10.7717/peerj.4139

**Published:** 2017-12-21

**Authors:** Qianying Zhang, Kanchun Dai, Laixi Bi, Songfu Jiang, Yixiang Han, Kang Yu, Shenghui Zhang

**Affiliations:** 1 Department of Hematology, The First Affiliated Hospital of Wenzhou Medical University, Wenzhou, China; 2 Central Laboratory, The First Affiliated Hospital of Wenzhou Medical University, Wenzhou, China; 3 Division of Clinical Research, The First Affiliated Hospital of Wenzhou Medical University, Wenzhou, China

**Keywords:** Acute myeloid leukemia, Pretreatment platelet count, Overall survival, Disease-free survival

## Abstract

**Background:**

Pretreatment platelet count has been reported as a potential tool to predict survival outcome in several solid tumors. However, the predictive value of pretreatment platelet count remains obscure in *de novo* acute myeloid leukemia (AML) excluding acute promyelocytic leukemia (M3).

**Methods:**

We conducted a retrospective review of 209 patients with *de novo* non-M3 AML in our institute over a period of 8 years (2007–2015). Receiver operating characteristic (ROC) curve analysis was used to determine the optimal platelet (PLT) cutoff in patients. We analyzed the overall survival (OS) and disease free survival (DFS) using the log-rank test and Cox regression analysis.

**Results:**

By defining the platelet count 50 × 10^9^/L and 120 × 10^9^/L as two cut-off points, we categorized the patients into three groups: low (<50 × 10^9^/L), medium (50–120 × 10^9^/L) and high (>120 × 10^9^/L). On univariate analysis, patients with medium platelet count had longer OS and DFS than those with low or high platelet count. However, the multivariate analysis showed that only longer DFS was observed in patients with medium platelet count than those with low or high platelet count.

**Conclusion:**

Our findings indicate that pretreatment platelet count has a predictive value for the prognosis of patients with non-M3 AML.

## Introduction

Acute myeloid leukemia (AML) is a malignant hematological neoplasm caused by the uncontrolled rapid proliferation of immature hematopoietic cells (Estey & Dohner, 2006; Lane, Scadden & Gilliland, 2009). As the disease progresses, the bone marrow hematopoietic function is impaired, which usually results in a decrease in number of circulating platelets derived from megakaryocytes. As we know, the principal function of platelets is to stop bleeding by clumping and clotting blood vessel injuries. Therefore, when platelet function remains normal, the maintenance of a certain number of platelets can avoid bleeding and significantly improve the prognosis of the patients.

As of today, the molecular and cytogenetic alterations of AML cells are being studied in detail (Dohner, Weisdorf & Bloomfield, 2015; Wierzbowska et al., 2017; Zhang et al., 2016). According to the national comprehensive cancer network (NCCN) guideline, cytogenetic subgroups are classified as favorable, intermediate, and unfavorable risk (O’Donnell et al., 2012). Basically, favorable risk included patients with t(8;21), inv(16), or t(16;16); unfavorable risk included patients with a complex karyotype (three abnormalities) or abnormalities of chromosome 5 and/or 7; and intermediate risk referred to patients with other findings, primarily a normal karyotype. However, the relation between complete blood count (CBC), an important examination of AML at diagnose and the prognosis of *de novo* AML patients excluding acute promyelocytic leukemia (M3) remains obscure. Platelet count at diagnose varies widely in individuals with AML (Lin et al., 2017; Rauch et al., 2016). The majority of patients have thrombocytopenia or severe platelet reduction at diagnosis, while a small number of patients have megakaryocytosis in bone marrow (BM), eventually leading to normal or even increased platelet count in peripheral blood (PB). Both thrombocytopenia and thrombocytosis are considered as risk factors of the prognosis in solid carcinomas. For example, in gastric carcinoma and nasopharyngeal carcinoma, thrombocytosis is considered to be an independent unfavorable factor for prognosis as it leads to a higher possibility of relapse or distant metastasis (Chen et al., 2015; Ikeda et al., 2002). Similarly, thrombocytopenia has been considered as an independent risk factor for prognosis in hepatocellular carcinoma (Wu et al., 2017). As to hematological malignancies, it has been reported that AML patients with DNMT3A mutations have a higher platelet count in PB and DNMT3A mutation has a correlation with poor prognosis (Thol et al., 2011). Additionally, several studies have also shown that in AML patients who underwent hematopoietic stem cell transplant (HSCT), persistent thrombocytopenia may be a poor prognostic factor for overall survival (OS), non-recurrence death and life-threatening infections (Bolwell et al., 2004; Kim et al., 2006; Zaja et al., 2011). Thus, although abnormal platelet number and function have been reported to occur in the development of leukemia (Glembotsky et al., 2014; Psaila et al., 2011), the prognostic impact of platelets in the total AML population is not well understood, especially the mutual relationship between pretreatment platelet count and prognostic characteristics including OS, disease-free survival (DFS) and poor response to therapy.

The aim of this retrospective study was to evaluate and discuss the prognostic value of platelet count at the initial time of diagnosis in a cohort of 209 newly diagnosed non-M3 AML patients. We found that patients with pretreatment platelet count in the range of 50–120 ×10^9^/L had a better prognosis than other patients.

## Patients and Methods

### Patients

In this retrospective study, we identified a total of 209 previously untreated patients with *de novo* non-M3 AML treated at the First Affiliated Hospital of Wenzhou Medical University from August 2007 to December 2015. Patients with preceding hematological disorders, therapy-related AML or with other carcinomas were excluded. All patients were aged between 14 and 60 years, received at least one course of induction chemotherapy with regular follow-up. The AML subtypes were diagnosed and classified based on the morphological, immunophenotypic, cytogenetic and molecular features of leukemic blast cells according to the French-American-British (FAB) and 2008 revision of the World Health Organization (WHO) classification of myeloid neoplasms (Vardiman et al., 2009). All procedures conformed to the Helsinki Declaration and the study was approved by the Institutional ethics committee (the IRB approval number is 2012-26).

All the patients received conventional induction chemotherapy (idarubicin 8–10 mg/m^2^ or daunorubicin 45–60 mg/m^2^ per day on days 1–3 or homoharringtonine 4–6 mg/m^2^ per day on days 1–7 and cytarabine 100 mg/m^2^ per day on days 1–7) after the time of initial diagnosis. There were 115 patients achieved complete remission (CR) in the first course of induction chemotherapy. Patients who did not achieve CR in the first course of induction chemotherapy received a second course of induction chemotherapy or salvage therapy. Finally there were 30 patients who failed to achieve CR at least two cycles of chemotherapy. Patients who achieved CR after one or two induction therapy received high-dose cytarabine-based consolidation. A total of 55 patients finally received allogeneic HSCT as postremission therapy.

### Cytogenetic analysis

Cytogenetic analysis of patient samples were performed in almost all patients at the time of initial diagnosis. BM samples were systematically examined for cytogenetic abnormalities by R- and/or G-banding techniques and classification according to the International System for Human Cytogenetic Nomenclature. At least 20 metaphases were observed in each sample.

### Statistical analysis

**Figure 1:**
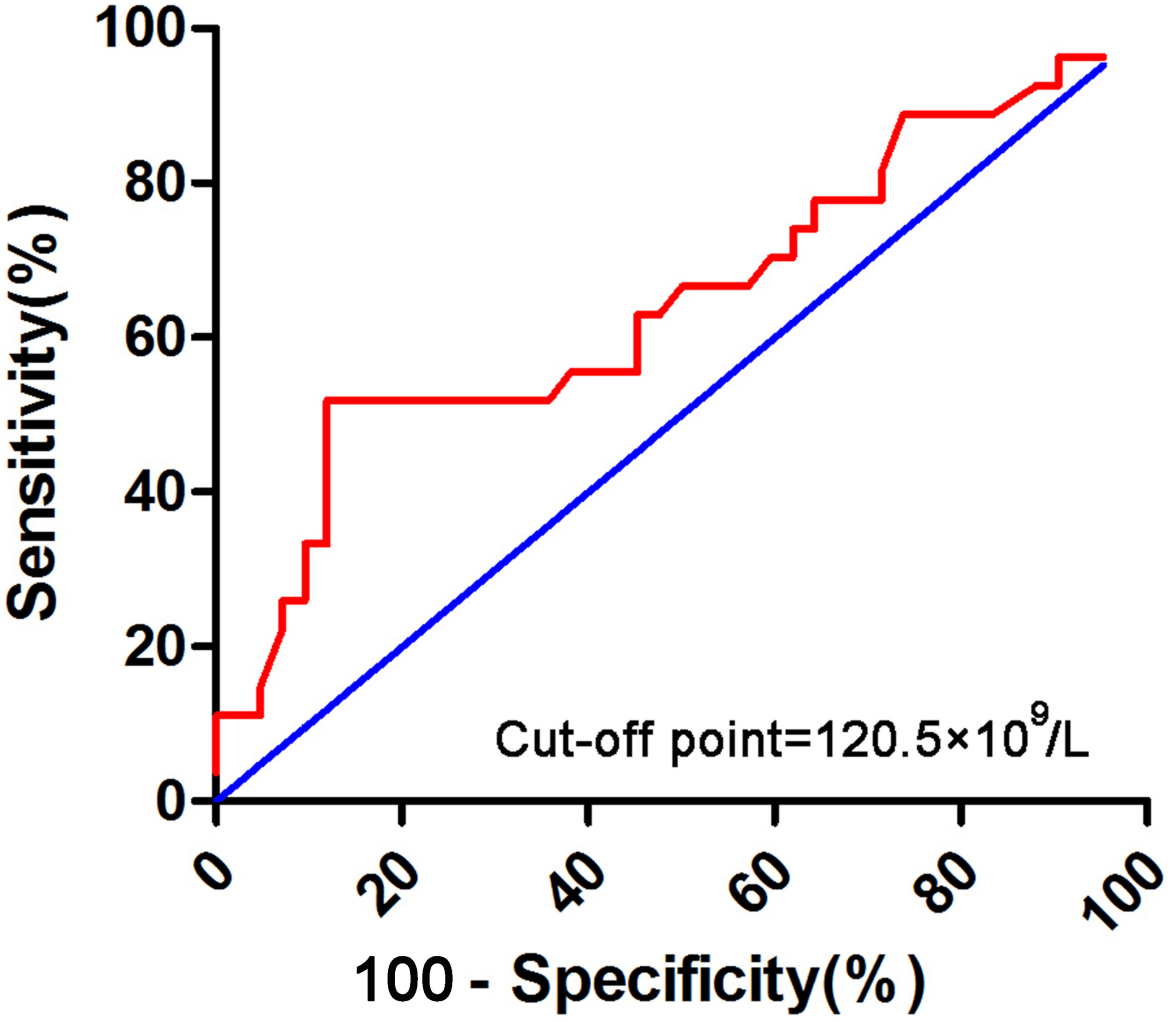
Receiver operating characteristic (ROC) curve analysis for initial PLT count over 50 × 10^9^/L.

Relations between platelet and recurrence rate were evaluated using the chi-square or Fisher’s exact test. Correlations among parameters observed in CBC were determined using Spearman’s rank correlation coefficient. OS was measured from the date of initial diagnosis to the date of death or last follow-up. DFS was measured from the date of CR to the time of relapse or death. Relapse was considered to have occurred when the BM contained 5% blasts unrelated to recovery from prior chemotherapy. OS and DFS were analyzed using Kaplan–Meier curves, which were compared using the log-rank test. Kolmogorov–Smirnov normality test was used to check the normality assumption for the data of baseline characteristics. Comparisons among patient clinical characteristics were done by Kruskal–Wallis test or Wilcoxon rank-sum test for continuous variables and Chi-square test or Fisher’s exact test for categorical variables. The vast majority of patients with newly diagnosed non-M3 AML in our cohort had thrombocytopenia, more than half of them were below 50 × 10^9^/L. Receiver operating characteristic (ROC) curve analysis was used to determine the optimal platelet (PLT) cutoff in patients with initial PLT count over 50 × 10^9^∕L. According to the ROC curve analysis, 120 × 10^9^/L was confirmed as the cut-off point of platelet count for survival outcome of AML (Fig. 1). Variables that were significant at *P* < 0.05 in the univariate Cox regression analysis were included in the multivariate analysis using forward stepwise selection. All tests were two-sided and a *P* value <0.05 was considered statistical significant. All statistical analyses were performed using SPSS software (ver. 17.0).

## Results

### Patient characteristics

A total of 209 non-M3 AML patients were included in our analysis and their general characteristics were summarized in Table 1. The median age was 40 years (range, 14–60 years), with 94 female and 115 male patients. The median white blood cell (WBC) count was 15.52 × 10^9^/L (range, 0.38–464 × 10^9^/L). The median percentages of blasts in PB and BM were 59% (range, 0–99%) and 69% (range, 9–98.8%), respectively. According to the FAB classification, there were two (0.96%) patients with M1, 36 (17.22%) patients with M2, 92 (44.02%) patients with M4, 66 (31.58%) patients with M5, seven (3.75%) patients with M6, and one (0.48%) patient with M7. The remaining five (2.39%) patients were unclassified. Cytogenetic analysis and/or molecular analysis of patient samples were performed in 207 patients at the time of initial diagnosis, in which 18, 171 and 18 patients showed favorable, intermediate, and unfavorable karyotypes, respectively. With a median follow-up of 21 months (range, 0–112 months), a total of 104 deaths were recorded, and the estimated 5-year OS and DFS were 40.3% (95% CI [31.5–49.1%]) and 33.3% (95% CI [24.3–42.3%]), respectively.

**Table 1:** Baseline patient characteristics.

Characteristics	All patients (*n* = 209)	Low initial PLT (<50 × 10^9^/L) (*n* = 125)	Medium initial PLT (50–120 × 10^9^/L) (*n* = 61)	High initial PLT (>120 ×10^9^/L) (*n* = 23)	*P* value
Median Age (range), years	40(14–60)	40(14–60)	40(17–59)	41(17–59)	0.976
Male/female	115/94	72/53	28/33	15/8	0.187
Median WBC count (range), ×10^9^/L	15.52(0.38–464)	25.2(0.38–464)	12.86(0.98–262.7)	6.55(1.19–129.56)	0.003
Median hemoglobin (range), g/L	76(32–153)	71(32–136)	81(41–127)	92(55–153)	0.001
Median platelets (range), ×10^9^/L	36(2–376)	22(2–50)	78(51–119)	147(122–376)	<0.001
Median blasts in PB (range),%	59(0–99)	70(0–99)	39(0–97)	20(0–84)	<0.001
Median blasts in BM (range),%	69(9–98.8)	70.4(12–98)	69.6(9–98.8)	61.20(23–97.5)	0.668
FAB subtypes, *n*(%)					
M0	0	0	0	0	–
M1	2(0.96)	1(0.80)	1(1.64)	0	1.000
M2	36(17.22)	27(21.60)	8(13.11)	1(4.35)	0.092
M4	92(44.02)	58(46.40)	24(39.34)	10(43.48)	0.675
M5	66(31.58)	30(24.00)	25(40.98)	11(47.82)	0.013
M6	7(3.35)	5(4.00)	2(3.28)	0	1.000
M7	1(4.78)	1(0.80)	0	0	1.000
Unclassified	5(2.39)	3(2.40)	1(1.64)	1(4.35)	1.000
Cytogenetic risk group, *n*(%)					0.822^a^
Favorable	18(8.61)	12(9.60)	5(8.20)	1(4.35)	–
Intermediate	171(81.82)	103(82.40)	51(83.61)	17(73.91)	–
Unfavorable	18(8.61)	9(7.20)	5(8.20)	4(17.39)	–
Missing	2(0.96)	1(0.80)	0	1(4.35)	–
Induction chemotherapy, *n*(%)					0.122
IA	190(90.91)	116(92.80)	55(90.16)	19(82.61)	–
DA	7(3.35)	3(2.40)	1(1.64)	3(13.04)	–
HA	12(5.74)	6(4.80)	5(8.20)	1(4.35)	–
CR^b^, *n*(%)	115(55.02)	71(56.8)	32(52.46)	12(50.00)	0.839
Relapse, *n*(%)	75(41.90)	49(44.55)	14(28.00)	12(63.15)	0.020
No. of patients who underwent HSCT, *n*(%)	55(26.32)	32(25.60)	20(32.79)	3(13.04)	0.178

**Notes.**

PLT platelet WBC white blood cell PB peripheral blood BM bone marrow FAB French-American-British HSCT hematopoietic stem cell transplant IA idarubicin and cytarabine DA daunorubicin and cytarabine HA homoharringtonine and cytarabine

a Comparison of the two cytogenetic subgroups (favorable versus other).

b Achieved complete remission (CR) after one course of induction therapy.

The median platelet count for all patients at diagnosis was 36 × 10^9^/L, with 59.81%, 29.19%, 11% patients showed low platelet count (<50 × 10^9^/L), medium platelet count (50–120 × 10^9^/L) and high platelet count (>120 ×10^9^/L), respectively. Patient characteristics grouped according to pretreatment platelet count were also summarized in Table 1. Patients with high platelet count tended to have lower WBC counts and higher hemoglobin levels than those with low or medium platelet count (*P* = 0.003 and *P* = 0.001, respectively). Blasts in PB also showed a significant difference among low, medium and high platelet count groups (*P* < 0.001). Patients with higher platelet count tended to have lower blasts in PB. There was a significant difference in the ratio in patients with relapse among three groups, and patients with high platelet count at diagnosis were more likely to relapse after they achieved CR (*P* = 0.020).

### Prognostic impact of platelet count at the time of diagnosis

**Figure 2:**
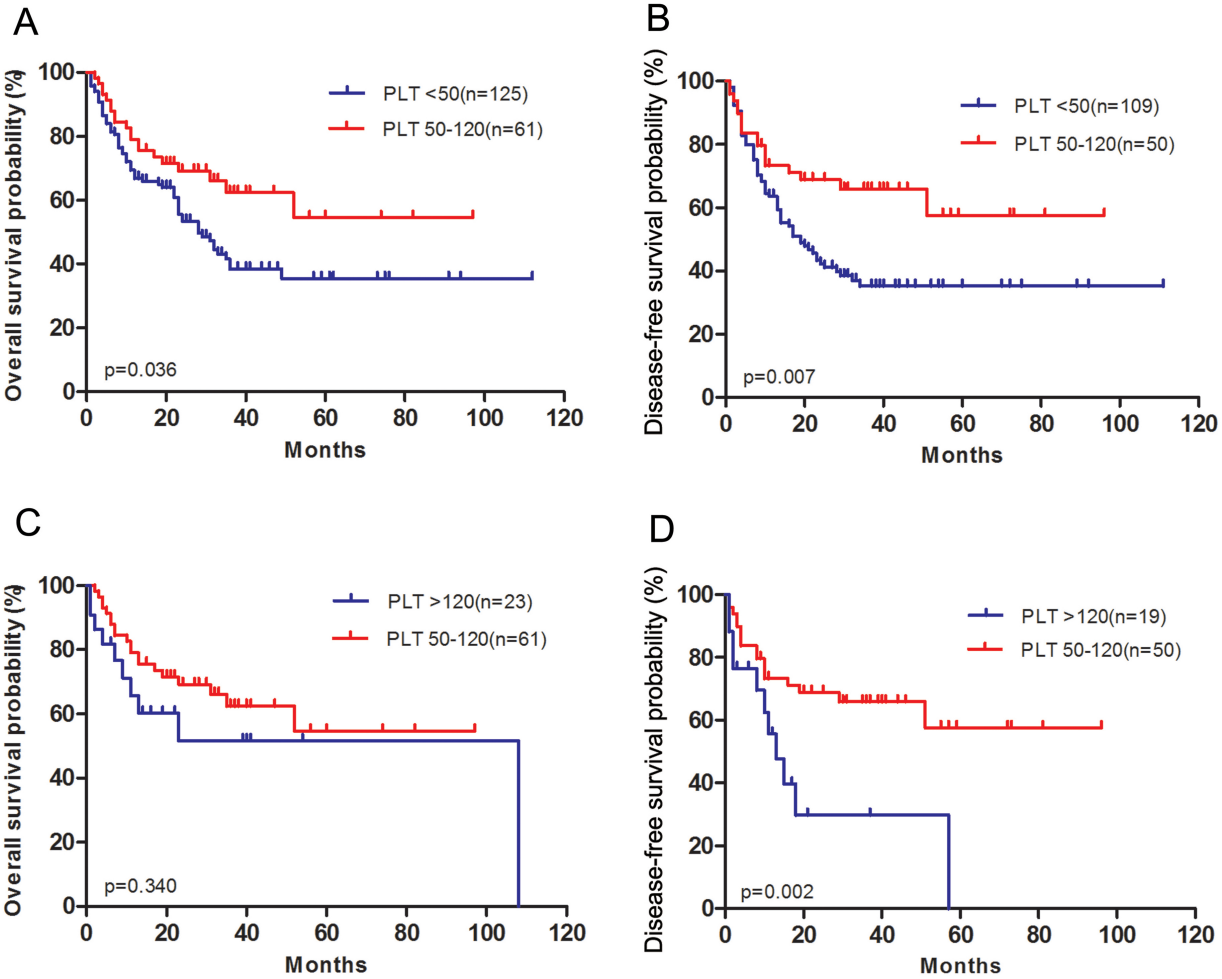
Survival outcomes of patients with acute myeloid leukemia grouped according to pretreatment platelet count. (A) Overall survival after diagnosis was compared between low platelet count group and medium platelet count group. (B) Disease-free survival after complete remission was compared between low platelet count group and medium platelet count group. (C) Overall survival after diagnosis was compared between medium platelet count group and high platelet count group. (D) Disease-free survival after complete remission was compared between medium platelet count group and high platelet count group.

Patients with medium platelet count achieved longer OS (*P* = 0.036, Fig. 2A) and DFS (*P* = 0.007, Fig. 2B) than those with low platelet count. There was no difference in OS (*P* = 0.340, Fig. 2C) using the Kaplan–Meier analysis between medium platelet group and high platelet group. However, patients with high platelet count had a significantly worse DFS compared to those with medium platelet count (*P* = 0.002, Fig. 2D).

The univariate Cox regression analysis was used to analyze the factors influencing OS and DFS in patients with AML. As shown in Table 2, the following clinical parameters were significantly associated with OS: platelets count (as a categorical variable), age, log(WBC) count and hemoglobin count (*P* = 0.044, *P* = 0.006, *P* = 0.006 and *P* = 0.019, respectively). The clinical parameters significantly associated with DFS were platelets (as a categorical variable) and age (*P* = 0.004 and *P* = 0.018, respectively).

**Table 2:** Univariate analyses for OS and DFS.

Characteristics	OS			DFS		
	OR	95% CI	*P* value	OR	95% CI	*P* value
Age (years)	1.022	1.006–1.038	0.006	1.019	1.003–1.036	0.018
Age (years, ≤40 vs. >40)	1.602	1.088–2.357	0.017	1.609	1.081–2.393	0.019
Gender	0.876	0.595–1.291	0.504	0.869	0.584–1.294	0.491
Log (WBC)	1.522	1.129–2.052	0.006	1.282	0.948–1.732	0.107
HB (g/L, <100 vs. ≥100)	1.929	1.113–3.343	0.019	1.596	0.943–2.700	0.082
PLT (×10^9^/L, continuous variable)	0.999	0.995–1.002	0.456	1.001	0.997–1.005	0.709
PLT (×10^9^/L, 50–120 vs. <50 or >120)	0.621	0.390–0.987	0.044	0.471	0.282–0.786	0.004
Blasts in PB (%, ≤20 vs. >20)	0.731	0.448–1.192	0.209	1.053	0.668–1.658	0.825
Blasts in BM (%, ≤50 vs. >50)	0.861	0.555–1.336	0.505	0.874	0.561–1.363	0.553

**Notes.**

HB hemoglobin PLT platelet PB peripheral blood BM bone marrow 95% CI 95% confidence interval

As presented in Table 3, the multivariate analysis included age, log(WBC), hemoglobin and platelets (as a categorical variable) as parameters revealed that age, log(WBC) and hemoglobin were significantly associated with OS (*P* = 0.004, *P* = 0.005, *P* = 0.013, respectively). Whereas platelets (as a categorical variable) failed to reach a statistical significance (*P* = 0.197). Additionally, age and platelets (as a categorical variable) were also significantly associated with DFS (*P* = 0.018, *P* = 0.013, respectively).

**Table 3:** Multivariate analysis of clinical factors for OS and DFS.

Characteristics	OS			DFS		
	OR	95% CI	*P* value	OR	95% CI	*P* value
Age (years)	1.023	1.007–1.040	0.004	1.019	1.003–1.036	0.018
Log (WBC)	1.529	1.135–2.060	0.005	1.254	0.930–1.691	0.137
HB (g/L,<100 vs. ≥100)	2.021	1.159–3.524	0.013	1.554	0.913–2.644	0.104
PLT (×10^9^/L, 50–120 vs. <50 or >120)	0.732	0.458–1.175	0.197	0.519	0.309–0.871	0.013

**Notes.**

HB hemoglobin PLT platelet 95% CI 95% confidence interval

**Figure 3:**
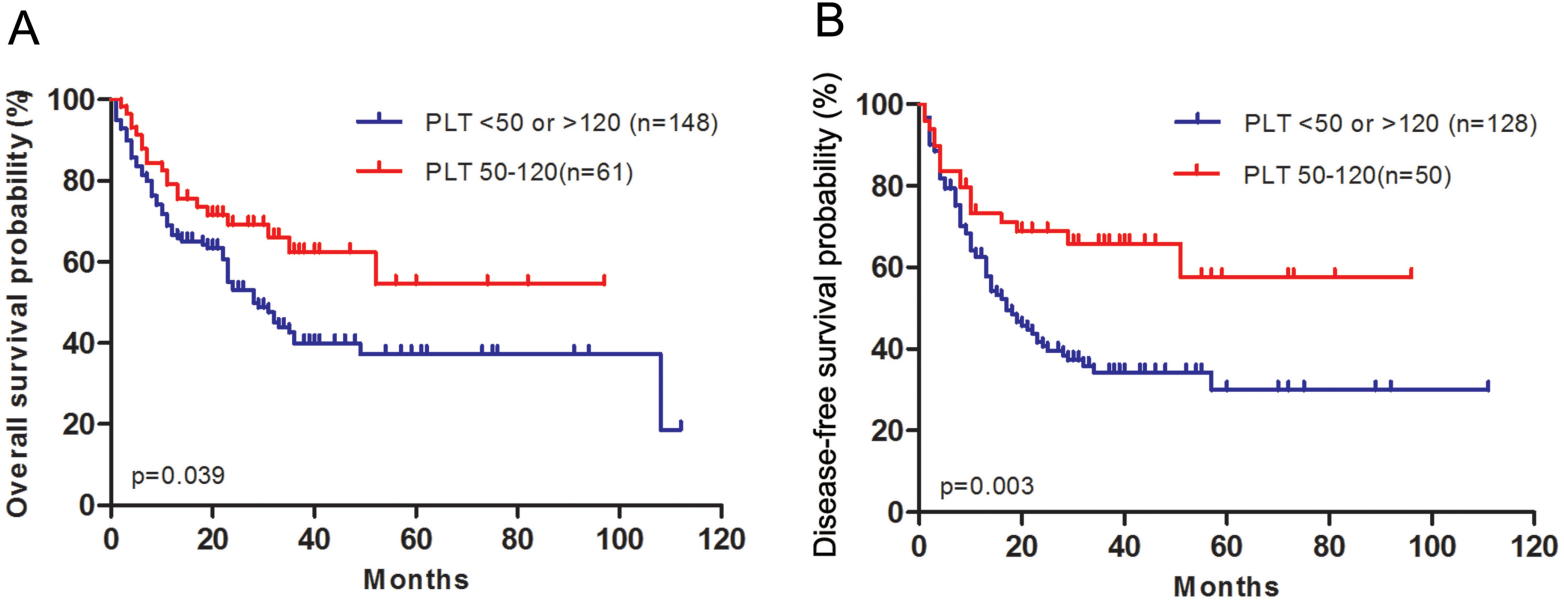
AML patients with medium platelet count have longer overall survival and disease-free survival than patients with low or high platelet count. (A) Overall survival after diagnosis was compared between patients with medium platelet count and patients with low or high platelet count. (B) Disease-free survival after complete remission was compared between patients with medium platelet count and patients with low or high platelet count.

Thus we divided all the patients into two groups, medium initial platelet count (50–120 × 10^9^/L) and low or high initial platelet count (<50 ×10^9^/L or >120 ×10^9^/L). The patient characteristics regrouped were shown in Table 4. Patients with medium platelet count had longer OS and DFS than patients with low or high platelet count (*P* = 0.039, *P* = 0.003, respectively, Fig. 3).

**Table 4:** Patient characteristics between medium initial PLT group and low or high initial PLT group.

Characteristics	Medium initial PLT (50–120 × 10^9^/L) (*n* = 61)	Low or high initial PLT (<50 or >120 × 10^9^/L) (*n* = 148)	*P* value
Median Age (range), years	40(17–59)	40(14–60)	1.000
Male/female	28/33	87/61	0.089
Median WBC count (range), ×10^9^/L	12.86(0.98–262.7)	18.48(0.38–464)	0.286
Median hemoglobin (range), g/L	81(41–127)	72.5(32–153)	0.070
Median platelets (range), ×10^9^/L	78(51–119)	24(2–376)	<0.001
Median blasts in PB (range), %	39(0–97)	68.5(0–99)	0.003
Median blasts in BM (range), %	69.6(9–98.8)	68.9(12–98)	0.537
CR^a^, *n*(%)	32(52.46)	83(56.08)	0.480
Relapse, *n*(%)	14(28.00)	61(47.29)	0.019
No. of patients who underwent HSCT, *n*(%)	20(32.79)	35(23.65)	0.173

**Notes.**

PLT platelet WBC white blood cell PB peripheral blood BM bone marrow FAB French-American-British HSCT hematopoietic stem cell transplant

a Achieved complete remission (CR) after one course of induction therapy.

In addition, a significant correlation was observed between platelet count and blast cells in PB (*P* < 0.001, Table 5). While the correlation between platelet count and blast cells in BM failed to reach a statistic significance (*P* = 0.515), similar to the results previously reported (Rauch et al., 2016).

**Table 5:** The correlations among parameters of patient characteristics.

Characteristics	Platelets (×10^9^/L)	
	Correlation coefficient	*P* value
WBC count (×10^9^/L)	−0.184	0.008
Hemoglobin (g/L)	0.265	<0.001
Blasts in PB (%)	−0.308	<0.001
Blasts in BM (%)	−0.043	0.533

## Discussion

In this retrospective study, we divided all the patients into three subgroups, as low, medium and high platelet count group (<50 ×10^9^/L, 50–120 × 10^9^/L, >120 ×10^9^/L, respectively). To our knowledge, this is the first study demonstrated pretreatment platelet count as an indicator to predict prognosis in AML patients. In those patients with an “almost normal” initial platelet count (>120 ×10^9^/L), we found that they have a higher relapse rate than others. Additionally, a shorter DFS was also observed in the high platelet count group.

Accordingly, we speculated that patients with the initial platelet count range from 50 × 10^9^/L to 120 × 10^9^/L may have a favorable clinical outcome than others. The possible causes are as follows: (1) patients in low platelet count group showed higher percentage of blasts in PB and lower hemoglobin, which mean severe myelosuppression or deeper infiltration in BM at the time of initial diagnosis, resulting in a poor recovery after induction chemotherapy (Miraki-Moud et al., 2013; Yamazaki et al., 2017); (2) although there was lesser blast cells in PB in high platelet count group than other two groups, it showed no difference in the blasts in BM. Platelets can release platelet growth factor acting on platelet-derived growth factor (PDGF) receptors on leukemia cells, which may affect the proliferation of leukemia cells (Foss & Bruserud, 2008). The effects caused by cytogenetic abnormalities may have an influence on the proliferation and differentiation of megakaryocytes, finally resulting in the increase of platelet count. Several studies have shown that some tumor cells can induce platelet aggregation, which is directly related to the ability to metastasize (Rickles, 2006; Tang & Honn, 1994). PDGF secreted by platelet has a strong activity in mitosis in cells, which can activate the DNA biosynthesis system and result in malignant proliferation in tumor cells (Ikeda et al., 2002).

Limitations still existed in our study. Firstly, it was a single-center and retrospective study, thus selection bias was difficult to be well balanced. For example, in contrast to the previously published population-based study, cases of M4 and M5 subtype were about 20–30% and about 10% in western countries (Byrd et al., 2002) (Palanisamy, 2010). While in our cohort it was 44.20% and 31.58%, respectively. Besides the population genetic diversity, the patient selection bias may be responsible for a particular FAB subtype of AML in our cohort. Thus, the prognostic role of pretreatment platelet count should be revalidated in the multivariate context including genetic information in the further prospective studies. Moreover, the bias caused by HSCT could not be ignored in the research. As there was no difference in the number of patients who underwent HSCT, we did not give an additional explanation. At last, the specific mechanism why and how platelets have an influence on the relapse of AML has not been explored in our study. We are going to have further research on molecular and cytogenetic levels.

In summary, this study demonstrated the relationship between platelet count at diagnosis and survival for non-M3 AML patients. Platelet count, as a common observed index in a routine CBC examination, may cause attention to us that it is a predictor of clinical outcomes in non-M3 AML patients. The correlations among platelet count, blasts in PB and hemoglobin were detected in our study, and no study has given a clear explanation to the interaction. These factors should be further analyzed in order to provide further information regarding our observation.

## Supplemental Information

10.7717/peerj.4139/supp-1 Supplemental Information 1 Raw Data

## Additional Information and Declarations

### Data Availability

The following information was supplied regarding data availability: The raw data is included in the  Supplemental Information.

## References

[ref-1] Bolwell B, Pohlman B, Sobecks R, Andresen S, Brown S, Rybicki L, Wentling V, Kalaycio M (2004). Prognostic importance of the platelet count 100 days post allogeneic bone marrow transplant. Bone Marrow Transplantation.

[ref-2] Byrd JC, Mrozek K, Dodge RK, Carroll AJ, Edwards CG, Arthur DC, Pettenati MJ, Patil SR, Rao KW, Watson MS, Koduru PR, Moore JO, Stone RM, Mayer RJ, Feldman EJ, Davey FR, Schiffer CA, Larson RA, Bloomfield CD (2002). Pretreatment cytogenetic abnormalities are predictive of induction success, cumulative incidence of relapse, and overall survival in adult patients with *de novo* acute myeloid leukemia: results from Cancer and Leukemia Group B (CALGB 8461). Blood.

[ref-3] Chen YP, Zhao BC, Chen C, Shen LJ, Gao J, Mai ZY, Chen MK, Chen G, Yan F, Liu S, Xia YF (2015). Pretreatment platelet count improves the prognostic performance of the TNM staging system and aids in planning therapeutic regimens for nasopharyngeal carcinoma: a single-institutional study of 2,626 patients. Chinese Journal of Cancer.

[ref-4] Dohner H, Weisdorf DJ, Bloomfield CD (2015). Acute myeloid leukemia. New England Journal of Medicine.

[ref-5] Estey E, Dohner H (2006). Acute myeloid leukaemia. Lancet.

[ref-6] Foss B, Bruserud O (2008). Platelet functions and clinical effects in acute myelogenous leukemia. Thrombosis and Haemostasis.

[ref-7] Glembotsky AC, Bluteau D, Espasandin YR, Goette NP, Marta RF, Marin Oyarzun CP, Korin L, Lev PR, Laguens RP, Molinas FC, Raslova H, Heller PG (2014). Mechanisms underlying platelet function defect in a pedigree with familial platelet disorder with a predisposition to acute myelogenous leukemia: potential role for candidate RUNX1 targets. Journal of Thrombosis and Haemostasis.

[ref-8] Ikeda M, Furukawa H, Imamura H, Shimizu J, Ishida H, Masutani S, Tatsuta M, Satomi T (2002). Poor prognosis associated with thrombocytosis in patients with gastric cancer. Annals of Surgical Oncology.

[ref-9] Kim DH, Sohn SK, Jeon SB, Baek JH, Kim JG, Lee NY, Suh JS, Lee KB, Shin IH (2006). Prognostic significance of platelet recovery pattern after allogeneic HLA-identical sibling transplantation and its association with severe acute GVHD. Bone Marrow Transplantation.

[ref-10] Lane SW, Scadden DT, Gilliland DG (2009). The leukemic stem cell niche: current concepts and therapeutic opportunities. Blood.

[ref-11] Lin CC, Hsu YC, Li YH, Kuo YY, Hou HA, Lan KH, Chen TC, Tzeng YS, Kao CJ, Chuang PH, Tseng MH, Chiu YC, Chou WC, Tien HF (2017). Higher HOPX expression is associated with distinct clinical and biological features and predicts poor prognosis in *de novo* acute myeloid leukemia. Haematologica.

[ref-12] Miraki-Moud F, Anjos-Afonso F, Hodby KA, Griessinger E, Rosignoli G, Lillington D, Jia L, Davies JK, Cavenagh J, Smith M, Oakervee H, Agrawal S, Gribben JG, Bonnet D, Taussig DC (2013). Acute myeloid leukemia does not deplete normal hematopoietic stem cells but induces cytopenias by impeding their differentiation. Proceedings of the National Academy of Sciences of the United States of America.

[ref-13] O’Donnell MR, Abboud CN, Altman J, Appelbaum FR, Arber DA, Attar E, Borate U, Coutre SE, Damon LE, Goorha S, Lancet J, Maness LJ, Marcucci G, Millenson MM, Moore JO, Ravandi F, Shami PJ, Smith BD, Stone RM, Strickland SA, Tallman MS, Wang ES, Naganuma M, Gregory KM (2012). NCCN clinical practice guidelines acute myeloid leukemia. Journal of the National Comprehensive Cancer Network.

[ref-14] Palanisamy N (2010). Chromosomal translocations in AML: detection and prognostic significance. Cancer Treatment and Research.

[ref-15] Psaila B, Bussel JB, Frelinger AL, Babula B, Linden MD, Li Y, Barnard MR, Tate C, Feldman EJ, Michelson AD (2011). Differences in platelet function in patients with acute myeloid leukemia and myelodysplasia compared to equally thrombocytopenic patients with immune thrombocytopenia. Journal of Thrombosis and Haemostasis.

[ref-16] Rauch PJ, Ellegast JM, Widmer CC, Fritsch K, Goede JS, Valk PJ, Lowenberg B, Takizawa H, Manz MG (2016). MPL expression on AML blasts predicts peripheral blood neutropenia and thrombocytopenia. Blood.

[ref-17] Rickles FR (2006). Mechanisms of cancer-induced thrombosis in cancer. Pathophysiology of Haemostasis and Thrombosis.

[ref-18] Tang DG, Honn KV (1994). Adhesion molecules and tumor metastasis: an update. Invasion and Metastasis.

[ref-19] Thol F, Damm F, Ludeking A, Winschel C, Wagner K, Morgan M, Yun H, Gohring G, Schlegelberger B, Hoelzer D, Lubbert M, Kanz L, Fiedler W, Kirchner H, Heil G, Krauter J, Ganser A, Heuser M (2011). Incidence and prognostic influence of DNMT3A mutations in acute myeloid leukemia. Journal of Clinical Oncology.

[ref-20] Vardiman JW, Thiele J, Arber DA, Brunning RD, Borowitz MJ, Porwit A, Harris NL, Le Beau MM, Hellstrom-Lindberg E, Tefferi A, Bloomfield CD (2009). The 2008 revision of the World Health Organization (WHO) classification of myeloid neoplasms and acute leukemia: rationale and important changes. Blood.

[ref-21] Wierzbowska A, Wawrzyniak E, Siemieniuk-Rys M, Kotkowska A, Pluta A, Golos A, Robak T, Szarawarska M, Jaskowiec A, Duszenko E, Rybka J, Holojda J, Grosicki S, Pienkowska-Grela B, Woroniecka R, Ejduk A, Watek M, Wach M, Mucha B, Skonieczka K, Czyzewska M, Jachalska A, Klonowska A, Iliszko M, Knopinska-Posluszny W, Jarmuz-Szymczak M, Przybylowicz-Chalecka A, Gil L, Kopacz A, Holowiecki J, Haus O (2017). Concomitance of monosomal karyotype with at least 5 chromosomal abnormalities is associated with dismal treatment outcome of AML patients with complex karyotype–retrospective analysis of Polish Adult Leukemia Group (PALG). Leukemia and Lymphoma.

[ref-22] Wu J, Hu G, Dong Y, Ma R, Yu Z, Jiang S, Han Y, Yu K, Zhang S (2017). Matrine induces Akt/mTOR signalling inhibition-mediated autophagy and apoptosis in acute myeloid leukaemia cells. Journal of Cellular and Molecular Medicine.

[ref-23] Yamazaki E, Kanamori H, Itabashi M, Ogusa E, Numata A, Yamamoto W, Ito S, Tachibana T, Hagihara M, Matsumoto K, Koharazawa H, Taguchi J, Tomita N, Fujimaki K, Fujita H, Fujisawa S, Ogawa K, Ishigatsubo Y (2017). Hyper-recovery of platelets after induction therapy is a predictor of relapse-free survival in acute myeloid leukemia. Leukemia and Lymphoma.

[ref-24] Zaja F, Geromin A, Patriarca F, Puglisi S, Cerno M, Sperotto A, Battista ML, Isola M, Fanin R (2011). Prognostic significance of delayed thrombocytopenia after allogeneic stem cell transplant. American Journal of Hematology.

[ref-25] Zhang YY, Huang SH, Zhou HR, Chen CJ, Tian LH, Shen JZ (2016). Role of HOTAIR in the diagnosis and prognosis of acute leukemia. Oncology Reports.

